# Supplementary material for: The role of spin in the degradation of organic photovoltaics
Source: Nat Commun. 2021 Jan 20;12:471. doi: 10.1038/s41467-020-20601-6 (PMC7817674; doi:10.1038/s41467-020-20601-6)
Supplement: Supplementary file 1 — Supplementary Information [file 41467_2020_20601_MOESM1_ESM.pdf]

Supplementary information for "The role of spin in the degradation of organic photovoltaics"

Ivan Ramirez[1], Alberto Privitera[2], Safakath Karuthedath[3], Anna Jungbluth[2], Johannes Benduhn[4], Andreas Sperlich[5], Donato Spoltore[4], Koen Vandewal[4†], Frédéric Laquai[3], Moritz Riede[2].

1. Heliatek GmbH, Treidlerstraße 3, 01139 Dresden 2. Clarendon Laboratory, Department of Physics, University of Oxford, Parks Road, OX1 3PU, Oxford, United Kingdom 3. King Abdullah University of Science and Technology (KAUST), KAUST Solar Center (KSC), Physical Sciences and Engineering Division (PSE), Thuwal 23955-6900, Kingdom of Saudi Arabia 4. Dresden Integrated Center for Applied Physics and Photonic Materials (IAPP) and Institute for Applied Physics, Technische Universität Dresden, Nöthnitzer Straße 61, 01187 Dresden, Germany, 5. Experimental Physics 6, Julius Maximilian University of Würzburg, Am Hubland, 97074 Würzburg, Germany, 6. *Institute for Materials Research (IMO-IMOMEC), Hasselt University, Wetenschapspark 1, 3590 Diepenbeek, Belgium*

Correspondance: ivan.ramirez@heliatek.com, moritz.riede@physics.ox.ac.uk

Supplementary Tables

Short name	Name	Structure	Supplier
α-6T	sexithiophene	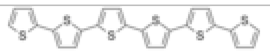	Lumtec
BPAPF	9,9-bis[4-(N,N-bis-biphyenyl-4-yl-amino)phenyl]-9H-fluorene	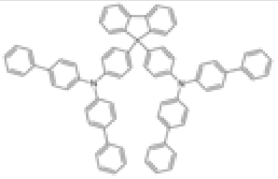	Lumtec
BPhen	4,7-Diphenyl-1,10-phenanthroline	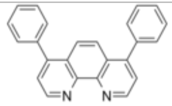	Lumtec
C_{60}	C_{60} fullerene	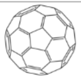	CreaPhys
DMFL-NPD	9,9-dimethyl-N,N'-diphenyl-N,N'-di-m-tolyl-9H-fluorene-2,7-diamine	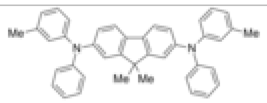	Lumtec
F_4ZnPc	Zinc(II)-1,8,15,22,tetrafluro-29H,31H-phtalocyanine	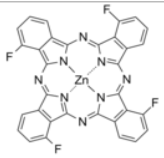	TU Dresden
MoO_3	Molybdenum trioxide		Lumtec
m-MTDATA	4,4',4'-'Tris(3-m-tolyl-phenylamino)triphenylamine	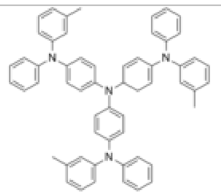	Lumtec
TAPC	1,1-bis[4-(N,N-di-ptolylamino)phenyl]cyclohexane	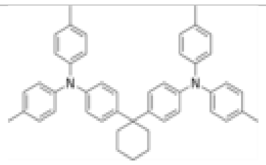	Sensient, Lumtec
TPDP	2,2',6,'6-tetraphenyl-4,4'-bipyranylidene	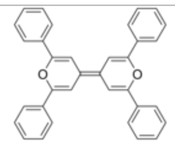	TU Dresden

Supplementary Table 1: material names and origin. All materials were at least once sublimed.

		α-6T:C_{60} (1:19)	TAPC:C_{60} (1:19)	F_4ZnPc:C_{60} (1:19)
Intersystem Crossing	*[D, E] (Mhz)*	[-168 22.5]	[-168 22.5]	[710 150]
	[p_x, p_y, p_z]	[0.4 0.6 0]	[0.4 0.6 0]	[0.3 0 1]
Back Hole Transfer	*[D, E] (MHz)*	[-168 22.5]	[-168 22.5]	-
	[p_+-p_0, p_--p_0]	[1 -1.07]	[1 -1.07]	-
	ISC:BHT weight	0.59:0.41	0.59:0.41	1.00:0.00

Supplementary Table 2: Best-fit values obtained for the TREPR spectra of α-6T:C_{60}, TAPC:C_{60} and F_4ZnPc:C_{60} (1:19). From the TREPR spectrum simulations, we obtain important information on the zero-field splitting (ZFS) parameters and the non-equilibrium populations of triplet sublevels (spin polarization), which are reported below. The ZFS parameters, *D* and *E*, define the magnetic dipolar interaction between the two electrons of the triplet state. The parameter *D*, in particular, defines the strength of the dipolar coupling and is strongly related to the delocalization of the triplet, while the parameter *E* represents the deviation of the triplet delocalization from axial symmetry.

Donor	E_{CT} /eV	*BHT*	*BET*	*FF*
TPDP	0.913[20]	No	No	
m-MTDATA	0.96	No	No	32
DMFL-NPD	1.28	No	No	33.2
TAPC	1.45	Yes	No	59.5
α-6T	1.5	Yes	No	53.7
BPAPF	1.55	Yes	No	45.3
F_4ZnPc	1.54	Yes	Yes	33.1

Supplementary Table 3: role of triplets in determining the FF. The FF does not correlate well with the presence of BHT deduced from dimerization/EPR. For F_4ZnPc BET is energetically possible and detected in TREPR experiment at 80K. The F_4ZnPc reorganization energy is for 1:1 blends.[28]

Supplementary Notes

Supplementary Note 1: BHT yield estimation

The UV-vis kinetics of figure 1c) of the main text are modelled as

$$N_{dim}(t) = N_0(1 - e^{-\frac{t}{\tau}})$$

where N_0 is the number of C_{60} molecules in the film, estimated from the quartz microbalance monitoring the deposition. The dimer to absorbed photon yield at early times is then given by

$$\Phi_{dim} = \frac{\frac{dN_{dim}}{dt}}{n_{abs}}(\mathrm{t} = 0) = \frac{N_0}{\tau \cdot n_{abs}}$$

where n_{abs} is the number of photons absorbed by the film in the infinitesimal time interval dt. This gives a photon to dimer yield of 1.1x10^{-4} for neat C_{60} and 4.9x10^{-5} for dilute TAPC:C_{60}. The dimer yield per absorbed photon can also be written as:

$$\Phi_{dim} = \Phi_{T1 \to dim}\Phi_{T1}$$

Where we assume the dimer per triplet yield $\Phi_{T1\rightarrow dim}$ to depend only on the temperature (topo-chemical effects) and morphology and therefore to be identical in all films.

The photon to triplet yield of neat C_{60} films, where dimerization occurs through ISC is given by:

$$\Phi_{T1}^{C60} = \Phi_{T1}^{ISC} = \frac{k_{ISC}}{k_{ISC} + k_{S1}}$$

The C_{60} neat film singlet recombination rate k_{S1} is known but the ISC rate is not (see supplementary note below and ref. [[1]]). We assume a time constant of 1 ns, close to the value in solution. By comparing Φ_{dim}^{C60} and Φ_{T1}^{C60} the triplet to dimer yield can be estimated. As this is independent of the triplet formation pathway provided the C_{60} morphology is not significantly affected, the BHT yield of TAPC:C_{60} dilute blends can then be obtained from $\Phi_{dim}^{TAPC:C60}$. The BHT rate

$$\Phi_{T1}^{BHT} = \frac{k_{BHT}}{k_{BHT} + k_{T1-CT} + k_{nr}} \approx \frac{k_{BHT}}{k_{BHT} + k_{T1-CT}}$$

is then related to the yield Φ_{T1} by the CT lifetime, which is reported in literature.[2]

Supplementary Note 2: Spin polarization of the C_{60} triplet state

When the donor does not absorb the pump beam (as is the case for TAPC, m-MTDATA and DMFL-NPD under 532 nm excitation) there are three possible pathways for the formation of C_{60} triplet states. For α-6T there is some residual donor absorption at 532 nm however, electron transfer is extremely efficient at dilute ratios meaning the below arguments also apply.

The first is intersystem crossing (ISC) of a singlet C_{60} exciton. This process occurs through a spin-orbit interaction and is characterized by a strong anisotropy of the populating rates of the three triplet sublevels (m_s=-1,0,+1). This anisotropy is responsible for the spin polarization of ISC-populated triplet states. ISC triplets can have several different spin polarization patterns, namely AAAEEE, EEEAAA, EAEAEA and AEAEAE, where A stands for enhanced absorption and E for emission.[5]

The alternatives involve back hole transfer (BHT) from the sublevels of the CT state possessing triplet character to the C_{60} triplet exciton, analogous to the more familiar back electron transfer (BET), in which a donor triplet state is formed from the CT state. The population of the triplet sublevels of C_{60} via BHT can originate either from the "spin correlated radical pair (SCRP) mechanism" or following non-geminate recombination of free charges.[5–7] In the SCRP mechanism, the standard recombination pathway starts from the hole transfer from $^1C_{60}$ to form a singlet ^{1}CT state, which in EPR spectroscopy is termed SCRP due to the strong magnetic interactions between the two unpaired spins of the CT state.[8,9] Unlike for closely bound excitons, the spin sublevels of an SCRP have mixed singlet and triplet character because of hyperfine and electron Zeeman interactions.[8] When a magnetic field is present, as in a standard TREPR experiment, in the first approximation only the m_s = 0 states of the SCRP possess a mixed singlet and triplet character. In this case, a spin-allowed recombination to the singlet ground state depletes the ms = 0 population leaving an excess population of SCRP T_{+1} and T_{-1} states. The SCRP T_{+1} and T_{-1} states can also undergo a spin-allowed BHT to an energetically low-lying neutral triplet state, followed by a slow spin-forbidden recombination to the ground state. This process generates an excess of spin population of the T_{+1} and T_{-1} C_{60} exciton states with a clear spin polarization pattern that can be either AEEAAE (D>0) or EAAEEA (D<0).[10,11] At zero magnetic field, i.e. during normal solar cell operation, the four SCRP levels are close in energy and mixed together mainly by the hyperfine

interaction so the intermixing occurs among all the four levels. The rate of intermixing in this case may be even faster than in standard X-band TREPR experiments but is unknown.

In the non-geminate recombination mechanism, since free charges are spin uncorrelated, by 'spin statistics' ¾ of events lead to the CT state sublevels with triplet character and ¼ to the ones with singlet character. Triplets generated by non-geminate recombination are not spin polarized and therefore cannot be detected in a standard TREPR experiment. As a matter of clarity, standard TREPR experiments of active layers of organic solar cells are commonly performed at low temperatures to slow down the spin relaxation processes and thus achieve a better signal-to-noise signal. The low temperature however also affects the photo-generation dynamics. Importantly TREPR is not sensitive to BHT originating from the non-geminate recombination of free charges as this process does not result in any spin polarization of the triplet sub-levels (the absorption and emission from each sub-level then cancels out). The presence of geminate BHT at high temperatures is still possible since magnetic interactions are present not only in the primary radical pair, which has a very short lifetime (several ps), but also in the following stages of charge separation. This could give the time to the radical pair to undergo spin-intermixing and therefore BHT to low-lying triplet states. The ratio of the rates between BHT occurring by SCRP and non-geminate mechanisms however is difficult to predict due to the many different processes involved and may vary significantly with temperature and from system to system.

Supplementary Note 3: Morphological changes upon dimerization

In neat C_{60}, the main morphological effect of oligomerization is a 0.7 % decrease in the lattice constant. As recently published by Moore et al, the average C_{60} coherence length obtained from GIWAXS for 6% mol TAPC:C_{60} is of circa 60 Å (about 5 C_{60} molecules)[12]. Thus the spacing at a C_{60} grain boundary will at most increase by about 2*(60 Å)*0.7% =0.8 Å. We do not believe this change (about half a C-C bond) allows for donor migration, especially considering that many of the donors used in our study are floppy. The invariance of the sensitive EQE CT bands upon dimerization also suggests that donor-C_{60} distance is unaltered and that the morphology is not significantly affected.

Supplementary Note 4: Effect of UV irradiation and temperature on interlayers

Ultraviolet (UV) irradiation is known to be damaging to OPV devices and in particular degrade interlayers.[13,14] The effect of the Xenon lamp UV radiation on devices must therefore be considered.

In our devices, BPhen/Al and MoO_3 contacts are used as these materials are both well-known and suitable for every used donor. A disadvantage is that both have known instabilities. BPhen is has a low glass transition temperature and tends to crystallise, which has only a small effect on J_{sc} provided the active layer is smooth.[15–17] MoO_3 is known to be UV-unstable and its photo-transformation has been thoroughly studied.[13] Crucially, significant spectral changes relating to MoO_3 only occur for λ>450nm (outside the spectral region of interest) and are only observed in the absence of ITO. Furthermore, in PHJs the MoO_3 interlayer was shown to not affect J_{sc} (pre-irradiation of the ITO/MoO_3 did not alter the degradation behaviour).[13] While these instabilities have consequences on the EQE magnitude they do not affect the spectral shape, from which we assess the progression of dimerization. Indeed, we find good agreement between absorbance (of neat films) and EQE data.

Supplementary Note 5: Reorganization energy

Given that BHT is an electron-transfer process, its rate can in principle be described by Marcus theory, according to which the BHT rate depends on E_{CT}, E_{T1} and the BHT reorganization energy λ_{BHT}. Yet, in figure 2, whether dimerization is observed can be predicted from just E_{CT} and E_{T1} without

considering λ_{BHT} (figure 2 and assuming $\lambda_{BHT} \approx \lambda_{CT}$ is primarily dictated by the donor).[19] This is likely because the observed quantity is the dimer yield, which is linked to the BHT triplet yield:

$$\Phi_{T1}^{BHT} = \frac{k_{BHT}}{k_{BHT}+k_{T1-CT}+k_{nr}} \approx \frac{k_{BHT}}{k_{BHT}+k_{T1-CT}} \qquad (1)$$

The rate of reverse hole transfer from T_1 to a triplet charge transfer state (^{3}CT) k_{T1-CT} and the (primarily non-radiative) CT recombination rate k_{nr} also play a role in this yield. k_{nr}has been studied in the context of V_{oc} losses and is well described by Marcus-Levich theory.[20] Near the sharp increase in dimer yield observed in figure 2, $E_{T1} - E_{CT} \ll E_{CT}$, which implies that k_{T1-CT} is much faster than k_{nr} (approx. in equation 1). Electron and hole transfer from singlet excitons to form CT states are known to be ultra-fast processes poorly described by Marcus theory for blends with fullerenes.[21–23] This is likely also true for triplets and means k_{T1-CT} will dominate equation (1) when it can occur. Thus Φ_{T1}^{BHT} will be small regardless of λ_{BHT} when $E_{CT} < E_{T1}$ and as such differences in λ_{BHT} do not play a significant role in determining the energetic cut-off at which dimerization occurs.

Supplementary Note 6: Dimer yield and exciton quenching

Because the dimerization rate is a first order process linear with illumination intensity, the following behaviour would be expected assuming that dimerization occurs through unquenched excitons, as originally supposed by Heumüller *et al.*[24]:

1. The dimerization yield depends on the exciton quenching yield.
2. The rate of dimerization is unaffected by the S_1→CT quenching rate (the remaining unquenched excitons react at the quenching rate seen in neat films). This is expected if the fullerene domain crystallinity (and therefore topo-chemical conditions) is relatively unchanged between the neat film and BHJ, as is the case for the dilute OPVs studied here.

We show below that this expected behaviour is not realised in our study: assuming that unquenched excitons contribute to dimerization by undergoing inter-system crossing (ISC) is inconsistent with the data.

The yield Φ_{ISC} can be calculated assuming first order processes:

$$\Phi_{ISC} = \frac{k_{ISC}}{k_{ISC} + k_{relax}}$$

where k_{ISC} is the ISC rate in neat C_{60} films and $k_{relax} = k_{HT} + k_{neat}$ is the S_1 relaxation rate, which proceeds primarily via hole transfer (HT) in blend films or non-radiative recombination of the S_1 in neat C_{60} films. We have previously shown k_{neat} to correspond to a 150 ps lifetime.[1]

If the rate of HT is not known, the IQE estimated from Transfer Matrix Modelling and EQE measurements (Supplementary figure 14) can provide a lower estimate for k_{HT}/k_{neat} and of the relative yield of unquenched S_1 excitons in the blend and neat films. To achieve for example an IQE of 90% in the C_{60} absorption region, an at least a ten-fold reduction in unquenched C_{60} excitons is required. Assuming that dimerization occurs through unquenched excitons, a ten-fold reduction in dimer yield and therefore in the change in absorption at 320 nm is required. This is clearly not observed (figure 1c of the main text).

It is possible to be more quantitative using the HT rates for each blend extracted from our TAS measurements and using the 150ps neat C_{60} value. As k_{ISC} is not precisely known in C_{60} *films*, a range of estimates is taken for the rate and used to calculate a range of possible Φ_{ISC} values. The k_{ISC} estimates are based around the values reported for C_{60} in solution (0.6-1.2ns) and the corresponding

Φ_{ISC} are plotted in Supplementary Figure 8.[25–27] From this figure, it can be seen that a very low ISC yield is expected for TAPC:C_{60} dilute blends - inconsistent with the high dimer yield found in Figure 1c) of the main text. The ISC yield is also higher for m-MTDATA:C_{60} than TAPC:C_{60} dilute blends (slower HT). Since a much higher dimer yield is observed the latter, ISC cannot be responsible for dimerization in dilute blends.

More generally, because HT is faster for TAPC:C_{60} than m-MTDATA:C_{60}, the above arguments also exclude any reaction mechanism that would occur prior to charge transfer and that correlates to unquenched excitons.

Supplementary Methods

JV measurements

JV characteristics were measured under simulated AM1.5g 100mW/cm2 sunlight generated by an A16S-003-300 (Solarlight Company Inc.) sun simulator with a Keithley 2400 sourcemeter. Calibration was performed at each run with a Si photodiode (S1337-33BQ, Hamamatsu, SR from Fraunhofer ISE CalLab, Freiburg).

The devices were not pre-conditioned prior to measurement. A measurement step of 20mV was used, with a 5ms settle time and 1ms sweep delay. The mismatch for dilute samples without donor absorption calculated to be 0.6112 and used for all samples, including F_4ZnPc for which some donor absorption occurs.

IQE estimation

IQE was estimated by comparing the experimental EQE to the absorption probability in the device active layer obtained from Transfer Matrix modelling.[3] The model was implemented in python and previously validated with transmission and reflection measurements.

Sensitive EQE measurements

The light of a quartz halogen lamp (50W) is chopped at 140Hz and coupled into a monochromator (Newport CS260) and stray light carefully minimized with additional long-pass filters. The resulting monochromatic light is focused onto the OSC; its current at short-circuit conditions is fed to a current pre-amplifier before it is analysed with a lock-in amplifier (Signal Recovery 7280 DSP). The time constant of the lock-in amplifier was chosen to be 1 s and the amplification of the pre-amplifier was increased to resolve low photocurrents. The normalised EQE_{PV} is determined by dividing the photocurrent of the OSC by the flux of incoming photons, which was obtained with a calibrated silicon (Si) and indium-gallium-arsenide (InGaAs) photodiode.

Transient absorption spectroscopy: further details

TA spectroscopy was carried out using a custom pump–probe setup.[4] The output of a titanium:sapphire amplifier (Coherent LEGEND DUO, 4.5 mJ, 3 kHz, 100 fs) was split into three beams (2, 1, and 1.5 mJ). Two of them were used to separately pump two optical parametric amplifiers (OPA) (Light Conversion TOPAS Prime). TOPAS 1 generates tunable pump pulses, while TOPAS 2 generates signal (1300 nm) and idler (2000 nm) only. TOPAS 2 was used to produce a white-light super continuum from 350 to 1100 nm by sending the 1300 nm pulses through a calcium fluoride (CaF2) crystal which is mounted on a continuously moving stage. For short delay TA measurements, TOPAS 1 was used to generate pump pulses, while the probe pathway length to the sample was kept constant at ≈5 m between the output of TOPAS 1 and the sample. The pump pathway length was varied between 5.12 and 2.6 m with a broadband retroreflector mounted on an automated

mechanical delay stage (Newport linear stage IMS600CCHA controlled by a Newport XPS motion controller), thereby generating delays between pump and probe from −400 ps to 8 ns.

For the 1 ns to 300 µs delay (long delay) TA measurement, the same probe white-light supercontinuum was used as for the 100 fs to 8 ns delays. Here the excitation light (pump pulse) was provided by an actively Q-switched Nd:YVO4 laser (InnoLas picolo AOT) frequency-doubled to provide pulses at 532 nm. The pump laser was triggered by an electronic delay generator (Stanford Research Systems DG535) itself triggered by the transistor– transistor logic (TTL) sync from the Legend DUO, allowing control of the delay between pump and probe with a jitter of roughly 100 ps.

The sample was kept under a dynamic vacuum of <10−5 mbar in a cryostat (Optistat CFV, OXFORD Instruments). The temperature-dependent study at 300-77 K was performed in a cryostat cooled by liquid nitrogen with various flow rates. A temperature controller (MercuryiTC, Oxford Instruments) was used to control the temperature. The transmitted fraction of the white light was guided to a custom-made prism spectrograph (Entwicklungsbüro Stresing) where it was dispersed by a prism onto a 512 pixel complementary metal-oxide semiconductor (CMOS) linear image sensor (Hamamatsu G11608- 512DA). The probe pulse repetition rate was 3 kHz, while the excitation pulses were mechanically chopped to 1.5 kHz (100 fs to 8 ns delays) or directly generated at 1.5 kHz frequency (1 ns to 300 µs delays), while the detector array was read out at 3 kHz. Adjacent diode readings corresponding to the transmission of the sample after excitation and in the absence of an excitation pulse were used to calculate $\Delta T/T$. Measurements were averaged over several thousand shots to obtain a good signal-to noise ratio. The chirp induced by the transmissive optics was corrected with a custom Matlab script. The delay at which pump and probe arrive simultaneously on the sample (i.e., zero time) was determined from the point of maximum positive slope of the TA signal rise for each wavelength.

MCR analysis notes

As a consistency check MCR was run on TA spectra obtained for C_{60} neat films. The MCR reproduces very well results from a recent study by some of us,[1] correctly identifying a fast component with mixed Frenkel and CT character ("CT exciton"), a slower singlet component ("S1 exciton") and at longer times a triplet component.

For simplicity we refer to the C_{60} excitons with mixed and pure Frenkel character as "singlets" when considering the dilute blends. Again, it is clear the MCR analysis does an excellent job of extracting the C_{60} exciton spectra for each blend (Supplementary Figure 10). Only minor variations in this signature are observed between blends, which we assign to the different time scales for HT at 77K (different balance of pure Frenkel and mixed character excitons) and minor variations in absorption spectra due to slight changes in morphology when different donors are used. We note that at low-T the mixed excitons are longer lived.

Supplementary figures

Possibility of back electron and back hole transfer

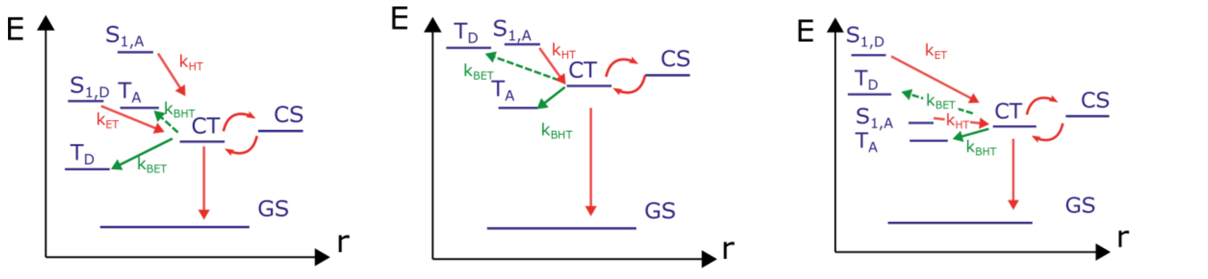

Supplementary Figure 1: common scenarios in which BET or BHT will dominate, illustrating the possibility of BET and BHT. Green arrows correspond to triplet formation pathways. Dashed arrows indicate suppressed pathways, Singlet and triplet states have been shifted in space for readability. BHT is the mirror process of BET, through which an acceptor triplet is formed instead of a donor triplet. The figure illustrates some common scenarios in a simplified fashion. On the left, a low-band gap donor with a fullerene acceptor and high S_1-CT driving force is illustrated. In this case, BHT is not possible but BET is. As most absorption occurs on the donor CT states are primarily formed from electron transfer (ET). The middle panel illustrates the TAPC, BPAPF, DMFL-NPD, m-MTDATA and diluted α-6T D:C_{60} blends used in this work, for which the donor is wide gap and BET is uphill. Here most absorption occurs on the fullerene. Hole transfer is the primary pathway and BHT is possible. Last, a narrow gap NFA with a complementary donor is shown (low driving force). In this case both ET and HT contribute significantly to the current but only BHT is possible. Another important case is illustrated in Figure 1c of the main text (donor absorption not shown), in which both BET and BHT are possible. This is relevant for example to green absorbers used in multi-junction devices.

Photo-degradation of C_{60} neat films on quartz (c.f. figure 1c of main text)

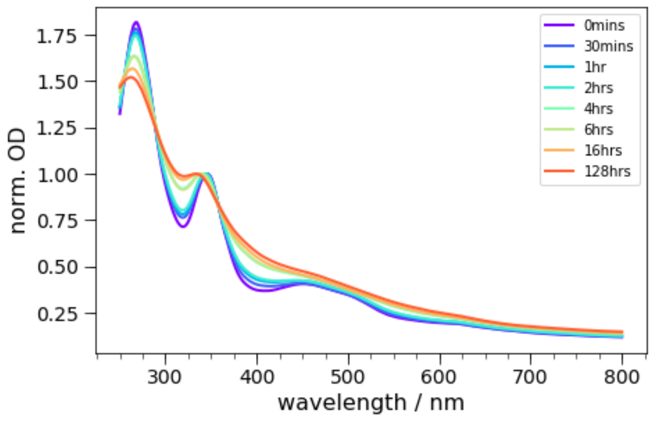

Supplementary Figure 2: evolution of UV-vis spectra of C_{60} films on quartz with exposure to white LED light. The evolution of the feature at 320 nm is used in the time series presented Figure 1 of the main text. The data has been normalised to the local peak at 350 nm.

Dimerization at higher donor ratios

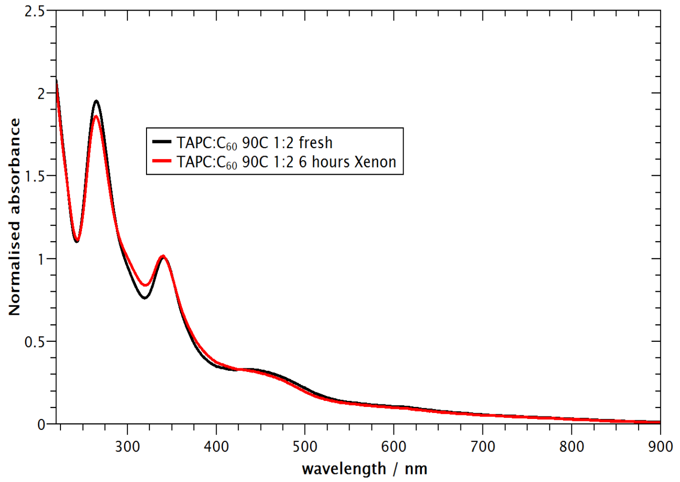

Supplementary Figure 3: evolution of UV-vis spectra of TAPC:C_{60} bulk heterojunctions processed at 1:2 volume (37% molar) and a substrate temperature of 90°C. These deposition conditions are representative of those employed with high efficiency donors. As for dilute blends, dimerization is evident.

Dimerization in F_4ZnPc:C_{60} dilute devices

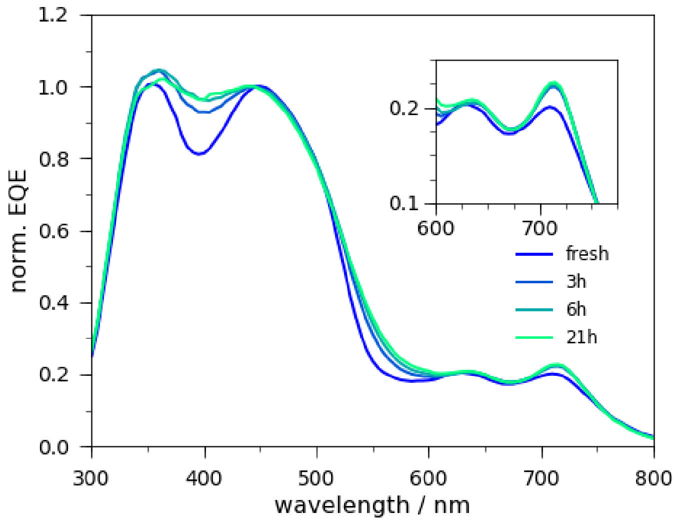

Supplementary Figure 4: evolution of normalised EQE spectra for an F_4ZnPc:C_{60} dilute blend device upon exposure to simulated sunlight (xenon lamp). Note that F_4ZnPc is not transparent to sunlight and contributes to the EQE in at 710 nm.

Temporal equivalence of UV-vis and EQE measurements

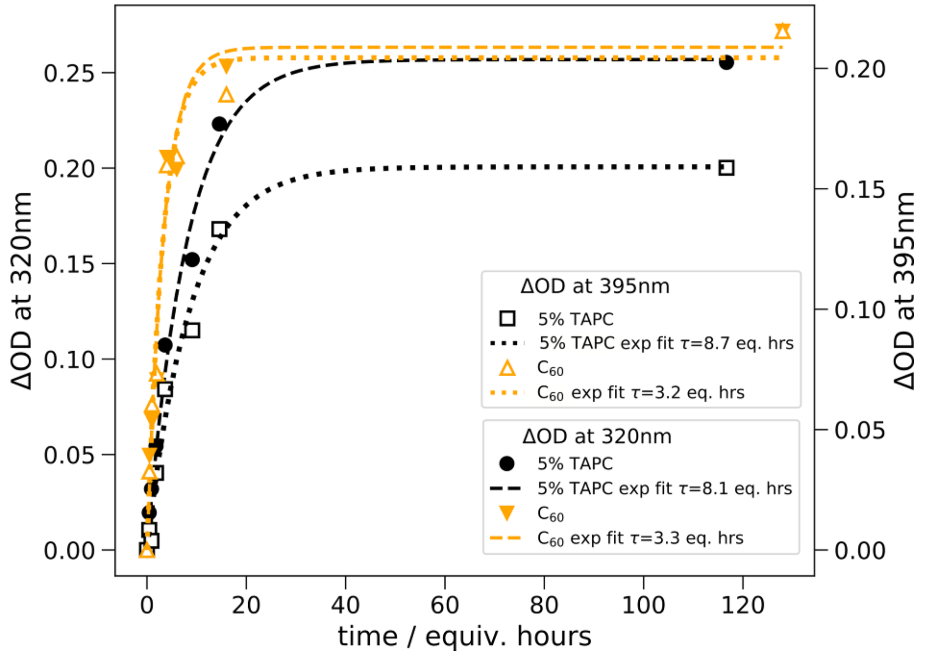

Supplementary Figure 5: Comparison of the change in UV-VIS absorption and exponential fit time constants at 320nm and 395 nm. Change in UV-VIS absorption data is presented for selected films on quartz a 320nm (dashed fit lines, solid symbols) and 395 nm (dotted fit lines, open symbols). The time axis has been scaled to compensate for the different film absorptions (C_{60} scaling = 1). For high C_{60} concentrations, the 320 and 395 nm are found to evolve at similar rates but with different amplitudes. A complication that arises from using the trough feature near 400 nm to monitor dimerization is that it's shape and therefore spectral position changes with light exposure considerably more than the trough at 320 nm. We expect this accounts for most of the mismatch in rates between the two tracking wavelengths.

Sensitive EQE measurements before and after light exposure (dimerization)

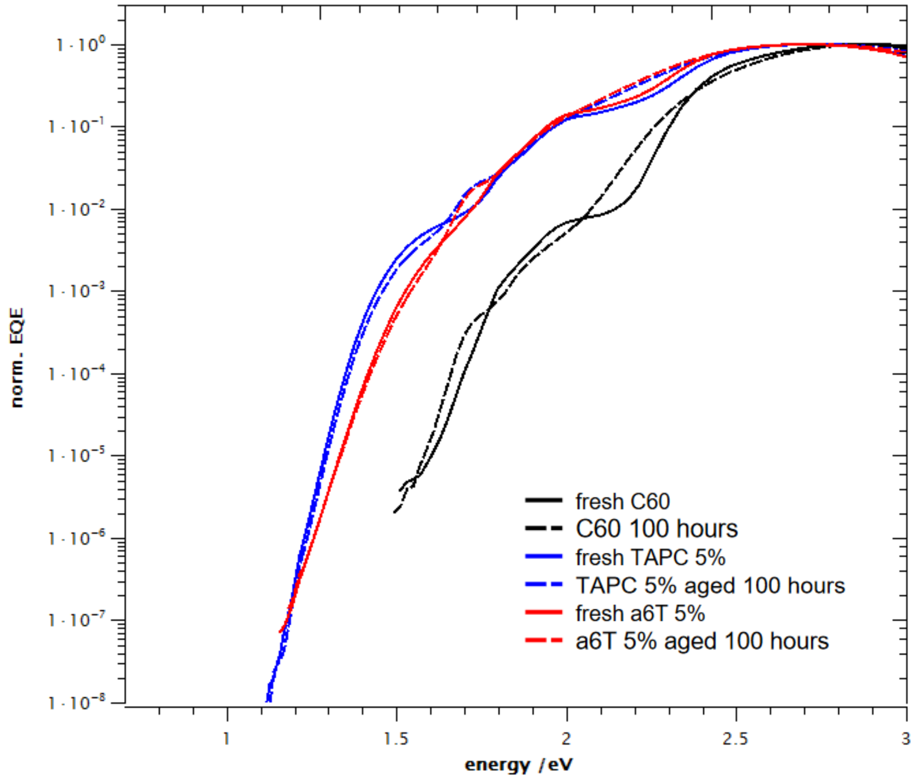

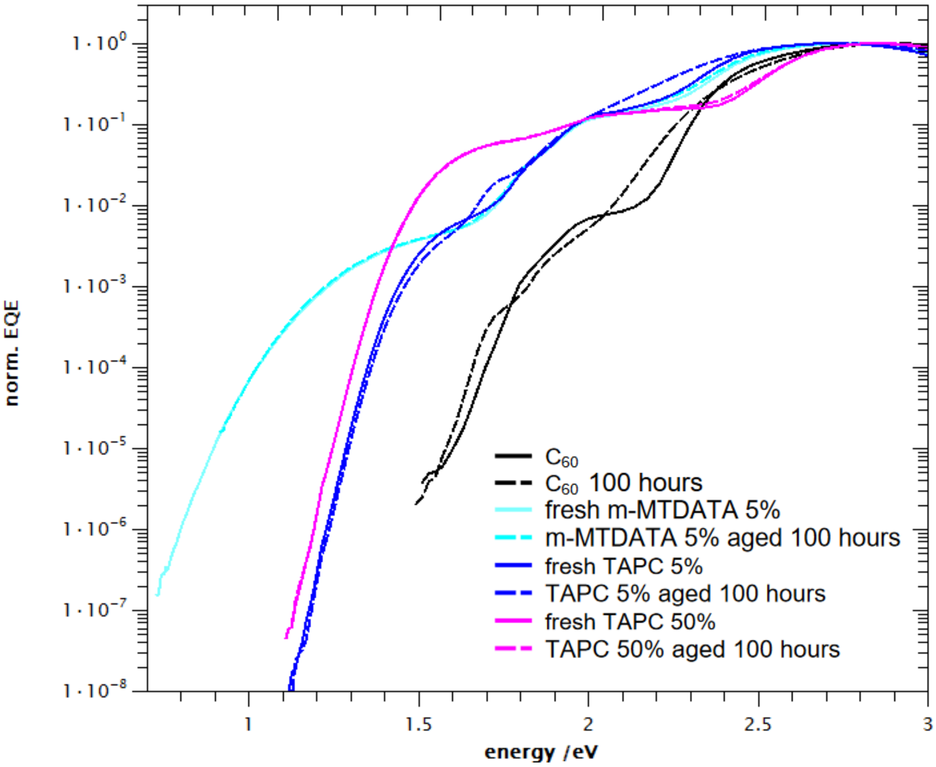

Supplementary Figure 6: sensitive EQE spectra before (solid lines) and after 100 hours exposure to simulated sunlight in a climate chamber at 65°C and 40% relative humidity (dashed lines).

TREPR Spectra for dilute $F_4ZnPC:C_{60}$ 1:19

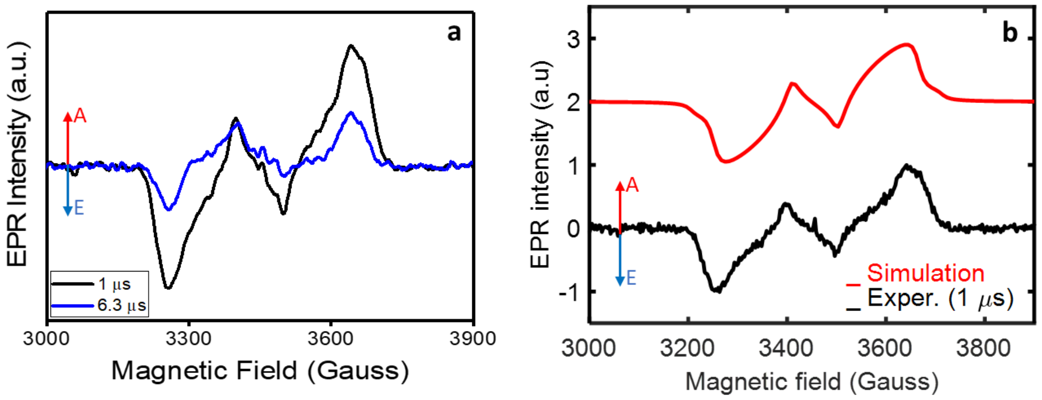

Supplementary Figure 7: (a) Smoothed TREPR spectrum at 80 K of $F_4ZnPc:C_{60}$ (1:19) recorded 1 µs (black line) and 6.3 µs (blue line) after a 532-nm laser pulse. (b) Best-fit spectral simulations (red line) of TREPR spectra (black line) at 1 µs. The values obtained from the simulation are reported in Supplementary Table 2. The TREPR spectrum of dilute $F_4ZnPC:C_{60}$ resembles that of an F_4ZnPC triplet with zero field splitting parameters D and E close to those reported in literature for the ZnPc triplet.[18] The spin polarization strongly deviates from an ISC triplet on ZnPc, indicative of a different triplet generation mechanism, namely the energetically favourable BET from an intermediate CT state. There is no clear contribution of C_{60} triplets in the TREPR spectrum. Though BET to F_4ZnPC has a much higher yield than C_{60} ISC, the absence of ISC signal could also be due to Dexter transfer from the C_{60} to F_4ZnPc triplet. This process is energetically favourable and likely pushes the signal below the detection threshold. We cannot quantitatively assess the relative contributions of BET and BHT+Dexter to the signal.

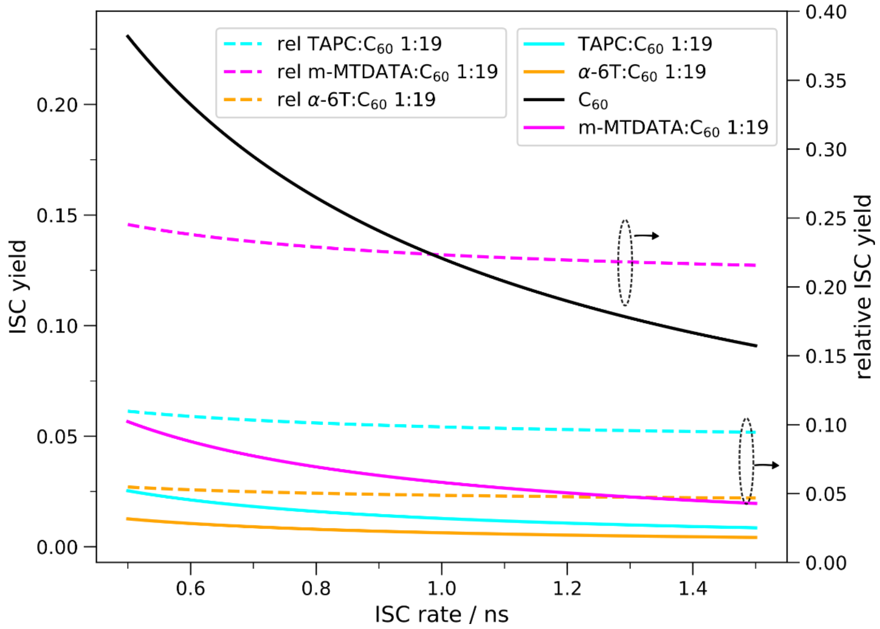

Supplementary Figure 8: calculated ISC yield at RT as function of assumed C_{60} ISC rate for various dilute blends and neat C_{60}. The right axis shows the blend yield relative to the neat C_{60} yield.

Transient absorption charge dynamics at 77K and 300K

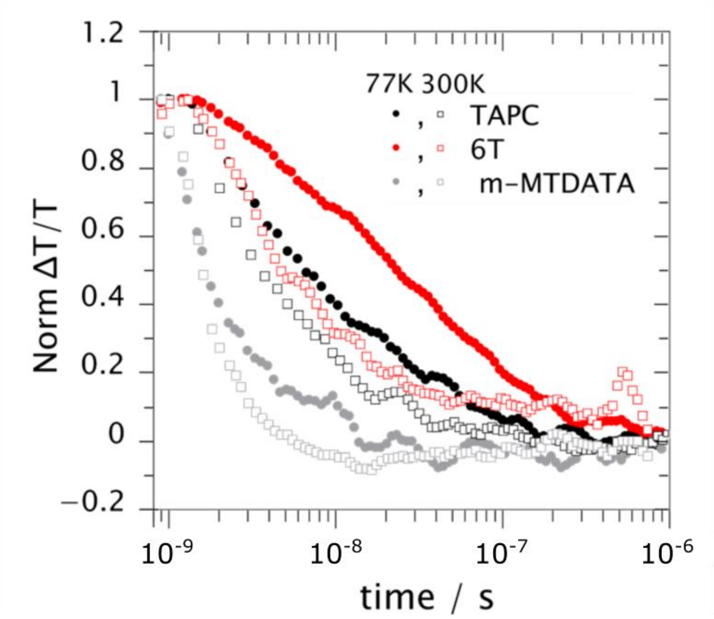

Supplementary Figure 9:, integrated remaining photo-absorption in the 1.8-2eV region for dilute blends with various donors at cryogenic (closed circles) and ambient temperatures (open squares) at ~30 µJ/cm^2

Picosecond-nanosecond TA spectra at 80K and RT used for the HT rate analysis

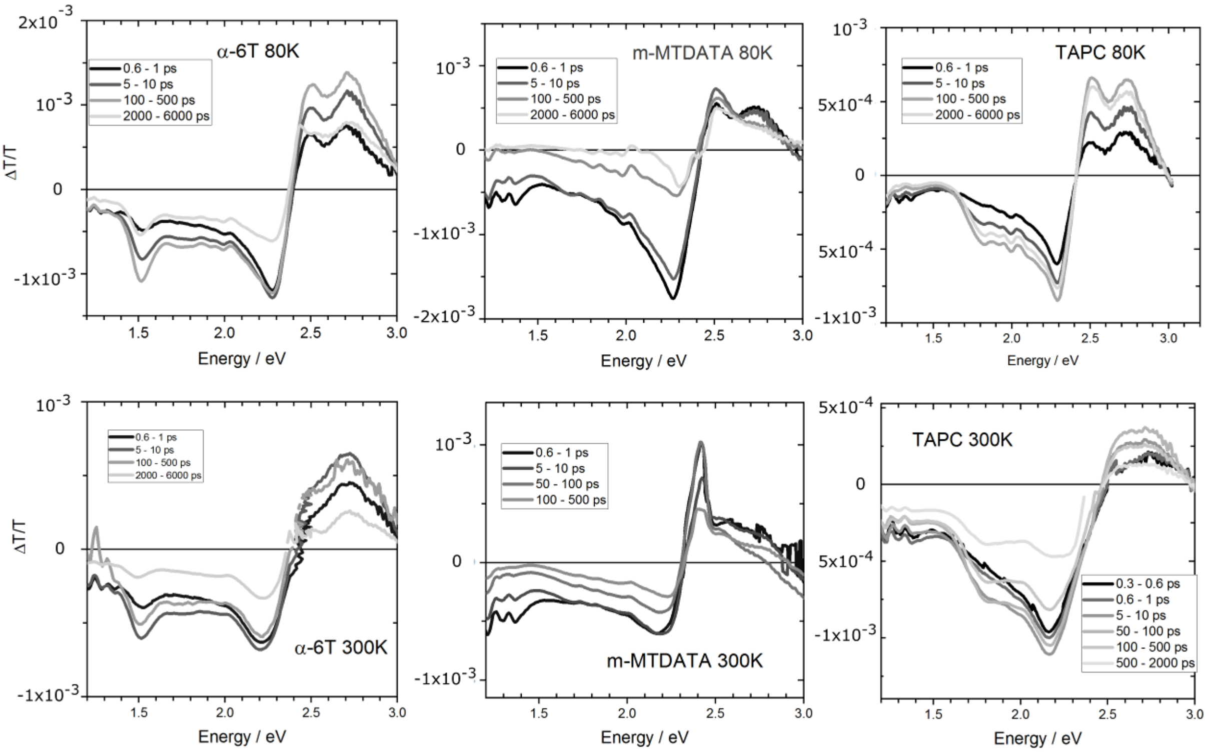

Supplementary Figure 10: ps-ns transient absorption spectra of dilute blends with various donors after excitation at 532 nm and at~40 µJ/cm^2. Top panels 80K, bottom panels 300K.

Component spectra obtained by MCR-ALS

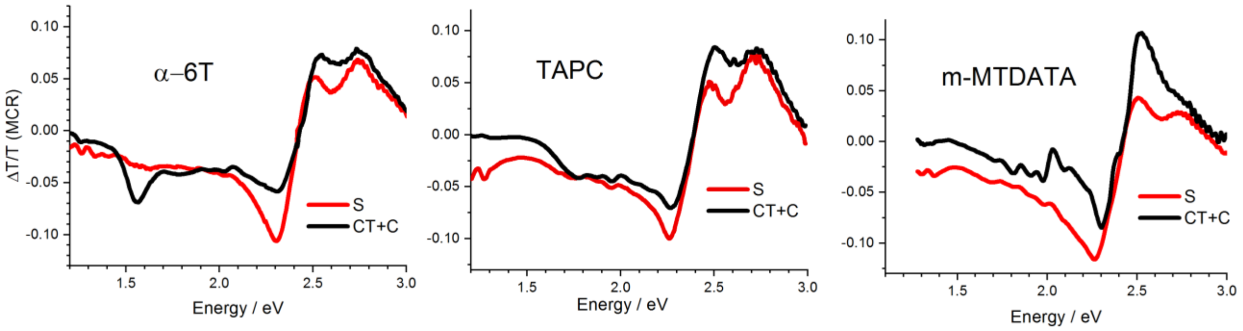

Supplementary Figure 11: MCR-ALS spectra of components contributing to the ps-ns TA data at 77K (neat C_{60} and comparison below). The m-MTDATA:C_{60} sample is 50 nm thick. The other dilute blends are 100 nm thick.

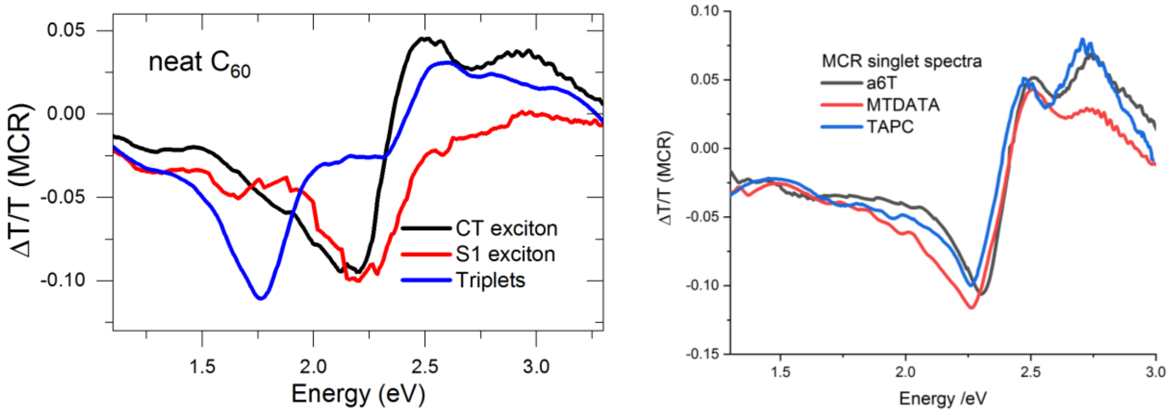

Supplementary Figure 12: left: MCR spectra for neat C_{60} at 300K. Right: comparison of the MCR singlet spectra for various dilute blends at 77K.

JV curves of the dilute blend devices and effect of BHT on FF

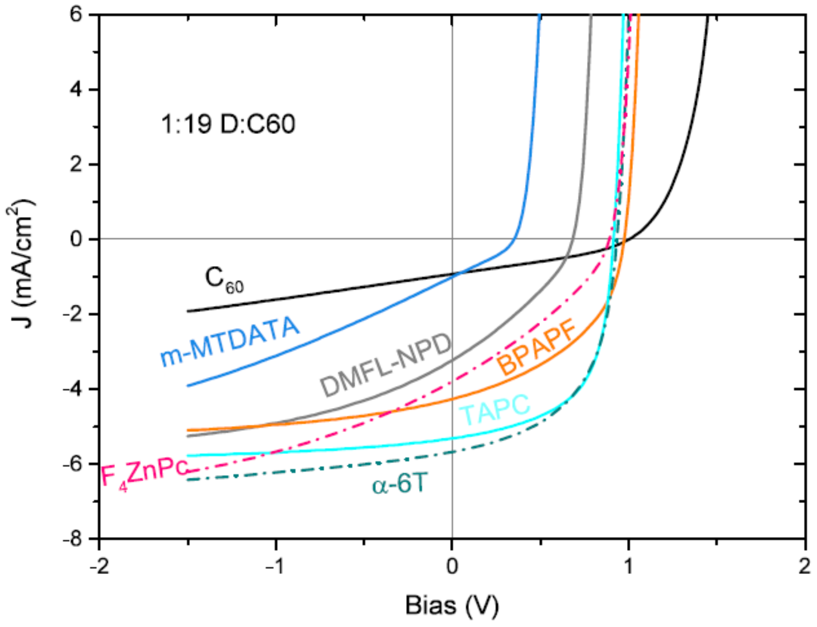

Supplementary Figure 13: initial performance of the dilute blend devices used for EQE degradation measurements, measured under 1 sun illumination. Solid lines indicate solar cells for which only C_{60} absorption contributes to the photo-current (*J* should coincide at very negative biases). Dashed dotted indicate solar cells in which the donor absorption is significant. A neat C_{60} device is included for reference. The *FF* has recently been demonstrated to depend primarily on the gap (V_{oc}) for dilute cells.[2] Note that F_4ZnPc has a surprisingly low *FF* for its V_{oc}. This could be due to its high BET rate.

IQE estimation for TAPC:C60 dilute blend devices

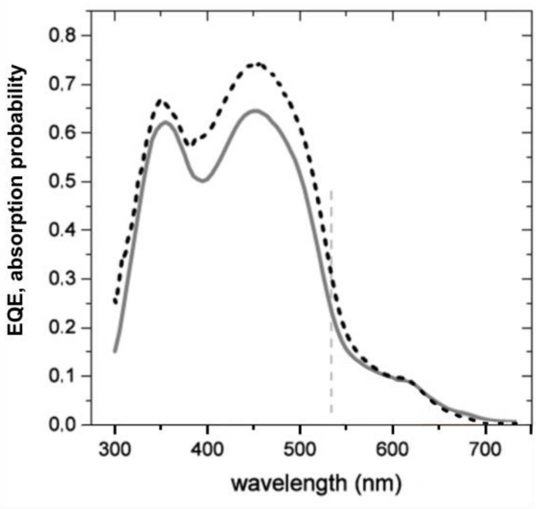

Supplementary Figure 14: EQE of TAPC:C_{60} 1:19 (measured, solid grey line) and probability of photon absorption in the active layer calculated from transfer matrix modelling. The dilute blend is assumed to have the same optical constants as C_{60}. The vertical dashed gray line illustrates the onset of neat C60 photo-conductivity found by Kazaoui *et al.*[29] The closeness of the dashed black and solid grey curves suggests a very high IQE, in the 85-95% region. The apparent wavelength dependence explained by the assumption that the blend has the same optical constants as C_{60} (a potentially higher generation efficiency closer to the cathode could also play a role).

SCLC measurements

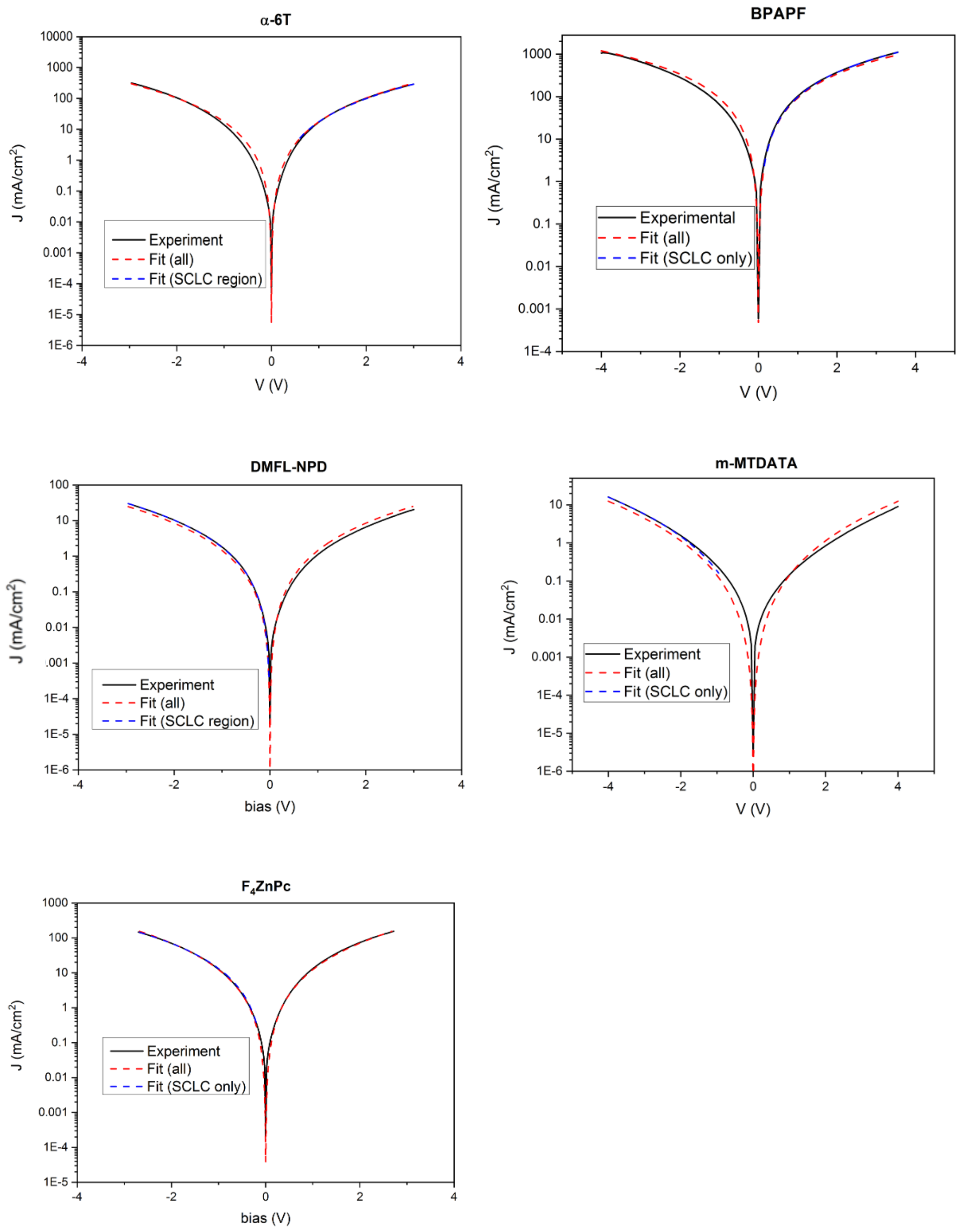

Supplementary Figure 15: SCLC curves measured for ITO/MoO_3/dilute blend (D:C_{60} 1:19)/MoO_3/Ag hole-only devices (solid lines) and fits according to $J = \frac{9}{8}\varepsilon_0\varepsilon_r\frac{V^2}{d^3}\mu_0 exp\left(\gamma\sqrt{V/d}\right)$ (dashed). Fit values are reported in Table 1 of the main text. All devices exhibit a slight asymmetry, which is particularly pronounced for BPAPF:C_{60} and related to injection at the contact.The data can be reasonably well fit over the whole range (red dashed lines) with $\varepsilon_r = 3.9 \pm 0.2$ and $d = 50 \pm 5$ nm. The values of Table 1 correspond to a sub-range at higher injection currents (blue dashed lines) and are close to those

previously reported in reference [30]. In all cases the error is dominated by the uncertainty in thickness (cubic). To ensure reliability, only devices with good forward and reverse bias symmetry were analysed. Furthermore, SCLC fits were only performed if a square-law voltage dependence was present at both forward and reverse biases for V> ±1-1.5V. While such a straightforward measurement scheme may not be sufficient for a precise determination of the mobility, it is sufficiently accurate to exclude mobility as a factor in dimerization (order of magnitude differences in the hole mobilities are found).

Supplementary references

1. Causa', M. *et al.* Femtosecond Dynamics of Photoexcited C_{60} Films. *J. Phys. Chem. Lett.* **9**, 1885–1892 (2018).

2. Collado-Fregoso, E. *et al.* Energy-Gap Law for Photocurrent Generation in Fullerene-Based Organic Solar Cells: The Case of Low-Donor-Content Blends. *J. Am. Chem. Soc.* **141**, 2329–2341 (2019).

3. Pettersson, L. A. A., Roman, L. S. & Inganäs, O. Modeling photocurrent action spectra of photovoltaic devices based on organic thin films. *J. Appl. Phys.* **86**, 487–496 (1999).

4. Karuthedath, S. *et al.* Charge and Triplet Exciton Generation in Neat $PC_{70}BM$ Films and Hybrid CuSCN:$PC_{70}BM$ Solar Cells. *Adv. Energy Mater.* **9**, 1802476 (2019).

5. Niklas, J. & Poluektov, O. G. Charge Transfer Processes in OPV Materials as Revealed by EPR Spectroscopy. *Adv. Energy Mater.* 1602226–n/a (2017). doi:10.1002/aenm.201602226

6. Budil, D. E. & Thurnauer, M. C. The chlorophyll triplet state as a probe of structure and function in photosynthesis. *Biochim. Biophys. Acta - Bioenerg.* **1057**, 1–41 (1991).

7. Thomson, S. A. J. *et al.* Charge Separation and Triplet Exciton Formation Pathways in Small Molecule Solar Cells as Studied by Time-resolved EPR Spectroscopy. *J Phys Chem C Nanomater Interfaces* **121**, 22707–22719 (2017).

8. Hore, P. J., Hunter, D. A., McKie, C. D. & Hoff, A. J. Electron paramagnetic resonance of spin-correlated radical pairs in photosynthetic reactions. *Chem. Phys. Lett.* **137**, 495–500 (1987).

9. Buckley, C. D., Hunter, D. A., Hore, P. J. & McLauchlan, K. A. Electron spin resonance of spin-correlated radical pairs. *Chem. Phys. Lett.* **135**, 307–312 (1987).

10. Righetto, M. *et al.* Engineering interactions in QDs-PCBM blends: a surface chemistry approach. *Nanoscale* (2018). doi:10.1039/C8NR03520B

11. Franco, L. *et al.* Time-Resolved EPR of Photoinduced Excited States in a Semiconducting Polymer/PCBM Blend. *J. Phys. Chem. C* **117**, 1554–1560 (2013).

12. Moore, G. J. *et al.* Ultrafast Charge Dynamics in Dilute-Donor versus Highly Intermixed TAPC:C60 Organic Solar Cell Blends. *J. Phys. Chem. Lett.* **11**, 5610–5617 (2020).

13. Zhang, H. *et al.* Photochemical transformations in fullerene and molybdenum oxide affect the stability of bilayer organic solar cells. *Adv. Energy Mater.* **5**, 1–9 (2015).

14. Patel, J. B. *et al.* Effect of Ultraviolet Radiation on Organic Photovoltaic Materials and Devices. *ACS Appl. Mater. Interfaces* **11**, 21543–21551 (2019).

15. Wang, N., Yu, J., Zang, Y., Huang, J. & Jiang, Y. Effect of buffer layers on the performance of

organic photovoltaic cells based on copper phthalocyanine and C60. *Sol. Energy Mater. Sol. Cells* **94**, 263–266 (2010).

16. Burlingame, Q. *et al.* Reliability of Small Molecule Organic Photovoltaics with Electron-Filtering Compound Buffer Layers. *Adv. Energy Mater.* **6**, 1–11 (2016).

17. Song, B., Burlingame, Q. C., Lee, K. & Forrest, S. R. Reliability of mixed-heterojunction organic photovoltaics grown via organic vapor phase deposition. *Adv. Energy Mater.* **5**, 1–6 (2015).

18. Akiyama, K., Tero-Kubota, S. & Ikegami, Y. Time-resolved EPR observation of the short-lived excited triplet states of diamagnetic metallophthalocyanines in a rigid glassy matrix. *Chem. Phys. Lett.* **185**, 65–67 (1991).

19. Vandewal, K. *et al.* Absorption tails of donor:C_{60} blends provide insight into thermally activated charge-transfer processes and polaron relaxation. *J. Am. Chem. Soc.* **139**, 1699–1704 (2017).

20. Benduhn, J. *et al.* Intrinsic non-radiative voltage losses in fullerene-based organic solar cells. *Nat. Energy* **2**, 17053 (2017).

21. Jakowetz, A. C. *et al.* What Controls the Rate of Ultrafast Charge Transfer and Charge Separation Efficiency in Organic Photovoltaic Blends. *J. Am. Chem. Soc.* **138**, 11672–11679 (2016).

22. Unger, T. *et al.* The Impact of Driving Force and Temperature on the Electron Transfer in Donor-Acceptor Blend Systems. *J. Phys. Chem. C* **121**, 22739–22752 (2017).

23. Bakulin, A. A., Hummelen, J. C., Pshenichnikov, M. S. & Van Loosdrecht, P. H. M. Ultrafast hole-transfer dynamics in polymer/PCBM bulk heterojunctions. *Adv. Funct. Mater.* **20**, 1653–1660 (2010).

24. Heumüller, T. *et al.* Morphological and electrical control of fullerene dimerization determines organic photovoltaic stability. *Energy Environ. Sci.* **9**, 247–256 (2016).

25. Sension, R. J. *et al.* Transient absorption studies of carbon (C_{60}) in solution. *J. Phys. Chem.* **95**, 6075–6078 (1991).

26. Sension, J., Szarka, A. Z., Smith, G. R. & Hochstrasser, R. M. Ultrafast photoinduced electron transfer to C_{60}. **185**, 179–183 (1991).

27. Salazar, F. A., Fedorov, A. & Berberan-Santos, M. N. A study of thermally activated delayed fluorescence in C_{60}. *Chem. Phys. Lett.* **271**, 361–366 (1997).

28. Vandewal, K., Benduhn, J. & Nikolis, V. C. How to determine optical gaps and voltage losses in organic photovoltaic materials. *Sustain. Energy Fuels* **2**, 538–544 (2018).

29. Kazaoui, S. *et al.* Comprehensive analysis of intermolecular charge-transfer excited states in C_{60} and C_{70} films. *Phys. Rev. B* **58**, 7689–7700 (1998).

30. Spoltore, D. *et al.* Hole Transport in Low-Donor-Content Organic Solar Cells. *J. Phys. Chem. Lett.* **9**, 5496–5501 (2018).
