# Supplementary material for: The role of spin in the degradation of organic photovoltaics
Source: Nat Commun. 2021 Jan 20;12:471. doi: 10.1038/s41467-020-20601-6 (PMC7817674; doi:10.1038/s41467-020-20601-6)
Supplement: Supplementary file 3 — Reporting Summary [file 41467_2020_20601_MOESM3_ESM.pdf]

nature research

Corresponding author(s): *Double-blind peer review submissions: write DBPR and your manuscript number here instead of author names.*



# Solar Cells Reporting Summary

Nature Research wishes to improve the reproducibility of the work that we publish. This form is intended for publication with all accepted papers reporting the characterization of photovoltaic devices and provides structure for consistency and transparency in reporting. Some list items might not apply to an individual manuscript, but all fields must be completed for clarity.

For further information on Nature Research policies, including our data availability policy, see Authors & Referees.

## ü Experimental design

**Please check: are the following details reported in the manuscript?**

1. Dimensions

| Item | Yes | No | Details |
|---|---|---|---|
| Area of the tested solar cells | ☒ | ☐ | Stated in methods |
| Method used to determine the device area | ☒ | ☐ | Stated in methods |

2. Current-voltage characterization

| Item | Yes | No | Details |
|---|---|---|---|
| Current density-voltage (J-V) plots in both forward and backward direction | ☐ | ☒ | Hysterisis is not present for these OPV devices. |
| Voltage scan conditions<br>*For instance: scan direction, speed, dwell times* | ☒ | ☐ | SI - JV measurements section |
| Test environment<br>*For instance: characterization temperature, in air or in glove box* | ☒ | ☐ | Samples encapsulated, see methods |
| Protocol for preconditioning of the device before its characterization | ☒ | ☐ | SI - JV measurements section |
| Stability of the J-V characteristic<br>*Verified with time evolution of the maximum power point or with the photocurrent at maximum power point; see ref. 7 for details.* | ☐ | ☒ | No high efficiency claims are made/not relevant |

3. Hysteresis or any other unusual behaviour

| Item | Yes | No | Details |
|---|---|---|---|
| Description of the unusual behaviour observed during the characterization | ☐ | ☒ | No unusual behaviour in JV |
| Related experimental data | ☐ | ☒ | No unusual behaviour in JV |

4. Efficiency

| Item | Yes | No | Details |
|---|---|---|---|
| External quantum efficiency (EQE) or incident photons to current efficiency (IPCE) | ☒ | ☐ | Main text and SI |
| A comparison between the integrated response under the standard reference spectrum and the response measure under the simulator | ☐ | ☒ | No high efficiency devices are presented. |
| For tandem solar cells, the bias illumination and bias voltage used for each subcell | ☐ | ☒ | No tandem solar cells |

5. Calibration

| Item | Yes | No | Details |
|---|---|---|---|
| Light source and reference cell or sensor used for the characterization | ☒ | ☐ | SI - JV measurements. |
| Confirmation that the reference cell was calibrated and certified | ☐ | ☒ | Available on request. |

Calculation of spectral mismatch between the reference cell and the devices under test

- [x] Yes
- [ ] No

SI - JV measurements section

6. Mask/aperture

Size of the mask/aperture used during testing

- [ ] Yes
- [x] No

Measured without aperture (defined area/no high efficiency claims)

Variation of the measured short-circuit current density with the mask/aperture area

- [ ] Yes
- [x] No

not applicable

7. Performance certification

Identity of the independent certification laboratory that confirmed the photovoltaic performance

- [ ] Yes
- [x] No

No efficiency claims made. Low efficiency model solar cells used - not certified

A copy of any certificate(s)
*Provide in Supplementary Information*

- [ ] Yes
- [x] No

Not certified

8. Statistics

Number of solar cells tested

- [ ] Yes
- [x] No

No efficiency claims made & devices highly reproducible (vacuum processing).

Statistical analysis of the device performance

- [ ] Yes
- [x] No

As above

9. Long-term stability analysis

Type of analysis, bias conditions and environmental conditions
*For instance: illumination type, temperature, atmosphere humidity, encapsulation method, preconditioning temperature*

- [x] Yes
- [ ] No

Methods and SI
